# Driving HCV Elimination Through Dedicated Hospital‐Based Teams and Pathways: A Retrospective and Prospective Study

**DOI:** 10.1111/liv.70668

**Published:** 2026-05-07

**Authors:** Pietro Torre, Enrica Serretiello, Giuseppe Di Siervi, Emilia Vaccaro, Tommaso Sarcina, Mariano Festa, Veronica Folliero, Mario Masarone, Gianluigi Franci, Marcello Persico

**Affiliations:** ^1^ Internal Medicine and Hepatology Unit, Department of Medicine, Surgery and Dentistry Scuola Medica Salernitana, University of Salerno Salerno Italy; ^2^ UOS Microbiology and Virology, AOU San Giovanni di Dio e Ruggi D'aragona Salerno Italy; ^3^ Postgraduate School in Clinical Pathology and Clinical Biochemistry University of Salerno Salerno Italy; ^4^ Molecular Biology Unit, AOU San Giovanni di Dio e Ruggi D'aragona Salerno Italy; ^5^ Department of Medicine, Surgery and Dentistry, Scuola Medica Salernitana University of Salerno Salerno Italy

**Keywords:** elimination strategies, HCV elimination, hepatitis C, hospital screening, viral hepatitis

## 1 Introduction

The advent of direct‐acting antivirals (DAAs) has had a significant impact on hepatitis C, given that for several years now, nearly all infected individuals can be cured through a short and highly effective course of therapy. Despite this, the World Health Organization (WHO) goal of viral hepatitis elimination by 2030 seems plausible only in a minority of countries [1, 2]. In response to this, and against a backdrop of declining global treatment uptake, screening and improved linkage‐to‐care protocols represent pivotal interventions. Among the settings where screening is considered profitable are hospitals, both for inpatients and outpatients or in emergency departments, a practice that presents a unique and powerful opportunity to identify individuals who might otherwise remain undiagnosed [3, 4]. The identification of HCV‐Ab positivity through screening, however, is only the first step in HCV care, and the subsequent phases, from the confirmation of active infection to the initiation of treatment, are often characterized by a significant loss of patients [5, 6]. Nevertheless, in the hospital, if dedicated protocols are put in place, these steps can be condensed, making the process easier for both physicians and patients. In this study, we conducted a retrospective evaluation of the entire HCV cascade of care at our hospital, subsequently applying an improved, standardized, linkage‐to‐care protocol for actively infected patients.

## 2 Patients and Methods

This study was conducted at the University Hospital San Giovanni di Dio e Ruggi d'Aragona, in Salerno, Campania Region (Italy), covering the period from 2016 to 2023 for the retrospective phase, and starting from May 2025 for the prospective phase. For the retrospective phase, admissions data and information regarding patients tested for HCV‐Ab and HCV‐RNA were extracted from hospital information systems; the proportion of treated patients was determined by cross‐referencing the cohort of HCV‐RNA positive patients with the internal databases of DAA prescription centres of Salerno and its province. In the prospective phase, all samples positive for HCV‐Ab underwent HCV‐RNA reflex testing, that is performing HCV‐RNA on the same sample used for the detection of HCV‐Ab [7]. Upon identification, patients with active infection were real‐time reported through shared files to the staff of Internal Medicine and Hepatology Unit. Following a remote assessment of patient profile and blood tests, three physicians began to reach patients when appropriate, providing essential information about hepatitis C and emphasizing the need for antiviral therapy. Department of origin was also considered, with a subdivision into Medical Departments (MDs), Surgical Departments (SDs) and Emergency Departments/Intensive Care Units (EDs/ICUs). Patient data were reported as absolute numbers and percentages or mean ± standard deviation (SD); associations between variables were assessed using the Chi‐square test and temporal trends were analysed with the Cochran–Armitage trend test. For all statistical tests, the significance level was set at an alpha value of 5%. Statistical analyses were performed using R software and GraphPad Prism.

## 3 Results

### 3.1 Retrospective Phase

**FIGURE 1:**
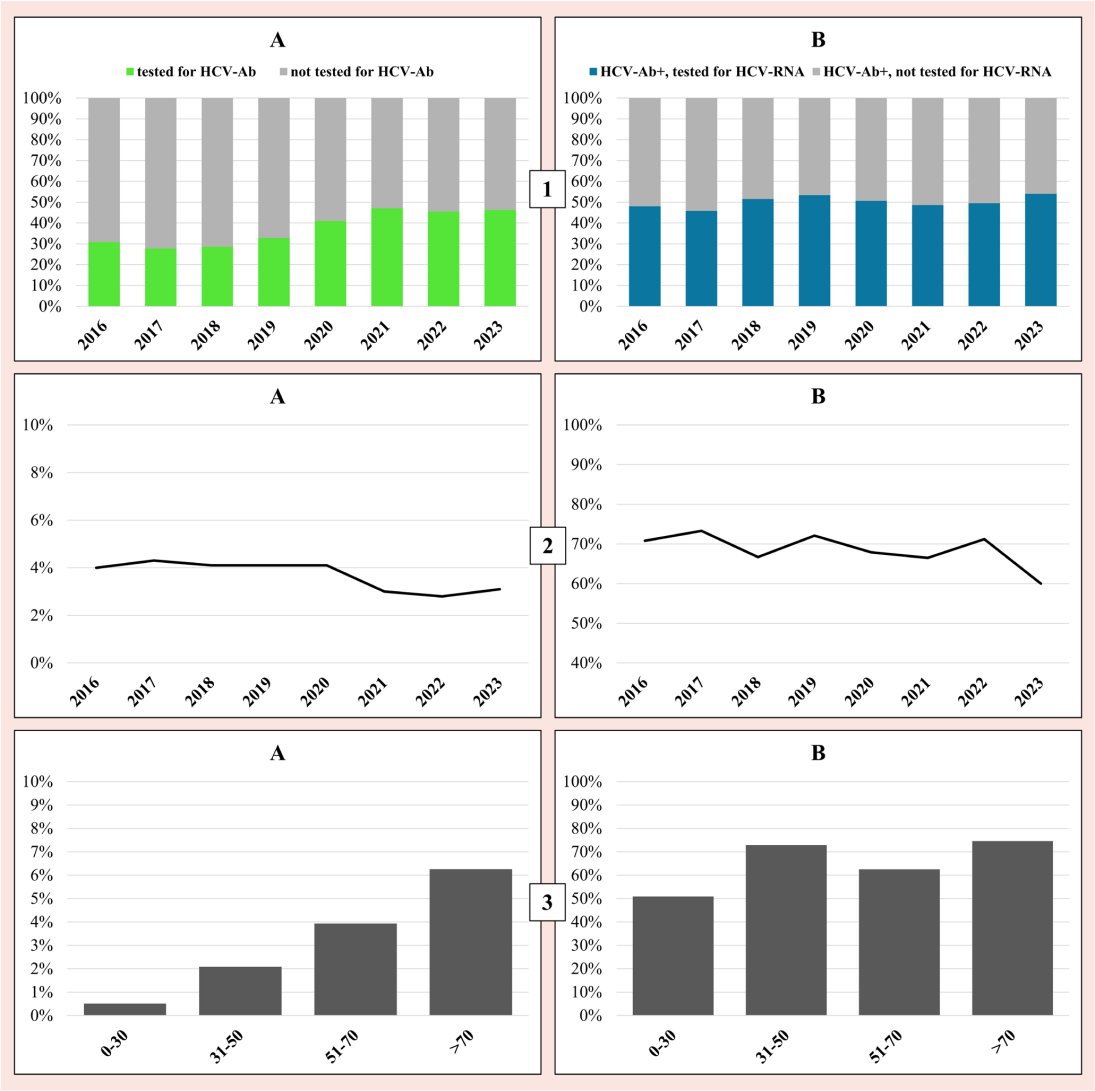
(1A) HCV screening in San Giovanni di Dio e Ruggi d'Aragona Hospital in Salerno, Campania Region (Italy), showing the share of HCV‐Ab testing among hospitalized patients during the period 2016–2023; (1B) HCV‐RNA testing among HCV‐Ab‐positive patients during the same period. (2A) Annual prevalence of HCV‐Ab‐positive patients, and (2B) annual prevalence of HCV‐RNA‐positive patients among those positive for HCV‐Ab during the period 2016–2023. (3A) Overall prevalence of HCV‐Ab‐positive patients by age group; (3B) overall prevalence of HCV‐RNA positivity by age group.

A total of 317 680 patients were admitted to the hospital between 2016 and 2023, including both ordinary and day hospital admissions. Among these, 116 478 (36.7%) underwent HCV‐Ab screening. Overall, HCV screening rates among hospitalized patients increased over the study period, with a significant variation over time, *p* < 0.001. Of the screened patients, 4214 tested positive, corresponding to an HCV‐Ab prevalence of 3.6%. Among positive patients, 2120 patients (50.3%) were tested for HCV‐RNA (*p* = 0.07 for trend over time), with the remaining half not being tested. 1450 (68.4%) were positive, while 670 (31.6%) tested negative. Testing rate (for HCV‐Ab and HCV‐RNA) over the years is shown in subfigures 1A and 1B of Figure 1.

Regarding HCV positivity over the years, a reduction in HCV‐Ab prevalence was found in more recent years, with approximately 4% in 2016–2020 and 3% in 2021–2023 (*p* < 0.001). The proportion of HCV‐RNA‐positive cases among individuals with HCV‐Ab showed a modest decline over time (*p* = 0.008). The percentage of positive subjects (for HCV‐Ab and HCV‐RNA) over the years is shown in subfigures 2A and 2B of Figure 1.

The mean age of HCV‐Ab‐positive subjects was 66.5 years (SD 16.4), and 2417 (57.4%) were male. HCV‐Ab prevalence increased with advancing age, peaking in patients aged > 70 at approximately 6% (*p* < 0.001). Actively infected patients had a mean age of 64 years (SD 15.6), and 916 (63.2%) were male. The highest prevalence of HCV‐RNA positivity was observed in the 31–50 and > 70 age groups (*p* < 0.001). The age of infected subjects is shown in subfigures 3A and 3B of Figure 1. Lastly, the proportion of HCV‐RNA‐positive patients who initiated DAA therapy was analysed. Approximately 60% of actively infected individuals received treatment, with non‐significant variation observed across the study period.

### 3.2 Prospective Phase

From May to November 2025, 385 HCV‐Ab‐positive patients were identified among 11 540 consecutively tested individuals, corresponding to a prevalence of 3.3%. Of these, all were tested for HCV‐RNA. Sixty‐eight patients (17.7% of HCV‐Ab‐positive patients and 0.6% of the prospectively tested cohort), with a mean age of 73 years and a male predominance, were found to be actively infected. Internal Medicine and Hepatology Unit recorded the highest number of HCV‐RNA positive cases. However, when considered collectively, SDs accounted for the highest overall number of HCV‐RNA‐positive cases. Furthermore, SDs and EDs/ICUs showed a higher share of active infection among HCV‐Ab‐positive patients compared to MDs (significant for SDs vs MDs, *p* = 0.0272). Fourteen patients died during hospitalization or shortly after discharge, six refused treatment, while the remaining 48 either received antiviral therapy, during their hospital stay or upon recall, or were scheduled for treatment initiation at the time of submission of the work.

Table 1 summarizes the findings of the retrospective phase and the characteristics of actively infected subjects identified during the prospective phase.

**TABLE 1:** HCV testing and patient characteristics.

Hospital HCV testing, retrospective phase (2016–2023)		
Hospital admissions, No.	317 680	
HCV‐Ab screening, No. (%)	116 478 (36.7)	
HCV‐Ab positive	No. (%)	4214 (3.6)
	Mean age (SD)	66.5 (16.4)
	Male gender, No. (%)	2417 (57.4)
HCV‐RNA testing/HCV‐Ab positive, No. (%)	2120 (50.3)	
HCV‐RNA positive	No. (%)	1450 (68.4)
	Mean age (SD)	64 (15.6)
	Male gender, No. (%)	916 (63.2)
HCV‐RNA‐positive patients, prospective phase (May–November 2025)		
Number	68	
Mean age, years (SD)	73.3 (14.2)	
Male gender, No. (%)	42 (61.8)	
Italian nationality, No. (%)	67 (98.5)	
Surgical Departments (SDs)	No. (%)	36 (52.9)
	% of active infection	22.0
Medical Departments (MDs)	No. (%)	23 (33.8)
	% of active infection	12.9
Emergency Departments/Intensive Care Units (EDs/ICUs)	No. (%)	9 (13.2)
	% of active infection	20.9

*Note:* HCV testing and characteristics of infected subjects identified during the retrospective phase of the study (2016–2023) and characteristics of HCV‐RNA positive subjects consecutively identified during the prospective phase at Ruggi d'Aragona Hospital in Salerno (the absolute number, the percentage relative to the total number of HCV‐RNA positive cases and the share of patients with active infection out of the HCV‐Ab‐positive subjects are shown across different types of hospital departments). Abbreviations: No., number; SD, standard deviation.

## 4 Discussion

In the context of decreasing treatment starts and Italy's transition from on‐track to off‐track status in the elimination process, hospitals represent a key opportunity for expanding HCV case‐finding and therapy, given the high concentration of older adults and patients with limited contact with the healthcare system who can be intercepted [2, 4]. Here, patient information, the tools needed to characterize liver disease (such as HCV‐RNA testing, liver stiffness measurement and ultrasound examination), and medical expertise are often colocalized. Several screening/linkage‐to‐care initiatives have been conducted in Italy in this setting, also in our region, demonstrating the feasibility and potential of such activity [8, 9].

Retrospective analysis of our study highlighted significant gaps in previous HCV care approaches, in the form of a lack of systematic and standardized protocols for hepatitis C. This translated, according to available data, into approximately 37% of people being tested for HCV‐Ab, and half of HCV‐Ab‐positive patients not being tested for HCV‐RNA, associated with about 40% of untreated HCV‐RNA‐positive patients. It is therefore evident that the intensification of screening efforts must necessarily be paired with active and coordinated hospital‐based interventions. During the prospective phase, the close collaboration between the involved Units improved the hepatitis C cascade of care, with all 385 HCV‐Ab positive patients identified in the 7‐month period tested for HCV‐RNA, and the 68 actively infected individuals systematically flagged and referred to our Hepatology Unit through computer systems and shared files. Following notification, three staff members of our Unit were responsible for reaching them to provide information and initiate treatment, either by visiting those still hospitalized or by recalling those who had already been discharged.

HCV‐Ab positivity remained comparable between the most recent years of the retrospective phase and the prospective phase, but the latter showed a reduced burden of active infection compared to previous years. This is consistent with the fact that our study did not exclude patients with a history of antiviral therapy, as reflex testing was performed automatically, and in parallel reflects the high volume of patients recently treated, accentuated by the expansion of treatment criteria in 2017. Significant findings also emerged regarding the department of origin: SDs had the highest number of HCV‐RNA‐positive patients, and SDs and EDs/ICUs registered a higher percentage of actively infected patients among the HCV‐Ab‐positive cohort compared to MDs. This is likely due to the characteristics of patients in such settings, which include individuals with presumably lower healthcare engagement and linkage‐to‐care, presenting for acute illnesses, emergencies and trauma and/or with specific HCV risk factors (such as a history of transfusions or parenteral exposure) [6, 10].

## 5 Conclusions

Hospitals represent an important site for HCV care, offering a unique opportunity to engage high‐risk and marginalized populations who may be unaware of or have neglected their infection over the years. Among the approaches to mitigate the burden on overworked hospitals and healthcare staff, especially those managing acute and life‐threatening conditions who may not have the time or expertise to deal with hepatitis C, the establishment of a dedicated hospital HCV team and pre‐established pathways is essential. These are necessary for managing all the phases that follow screening, ensuring it is not in vain but instead results in the treatment of infected patients. Implementing them on a national scale should be part of the proactive approach that is necessary at this historical moment of decreasing prevalence to reach the thousands of patients with still active infection and prevent their presentation at an advanced stage.

## Funding

The authors have nothing to report.

## Consent

The authors have nothing to report.

## Conflicts of Interest

The authors declare no conflicts of interest.

## Data Availability

The data that support the findings described here are available from the corresponding authors upon request.
